# The United States Goverment in Its Relations to Contagious Animal Diseases and the Veterinary Profession

**Published:** 1883-10

**Authors:** Frank S. Billings


### THE JOURNAL

OF

### COMPARATIVE MEDICINE AND SURGERY.

VOL. IV. OCTOBER, 1883. No. 4.

### ORIGINAL COMMUNICATIONS.

### ART. XXIV.—THE UNITED STATES GOVERNMENT IN ITS RELATION TO CONTAGIOUS ANIMAL DISEASES AND THE VETERINARY PROFESSION.

BY FRANK S. BILLINGS, V. S.

IN opening these questions we are obliged to look upon them from a political standpoint only. True politics is nothing else than the Science of Government. We see very little of this in the United States. If a stranger were to be asked what is the purpose of our Government, he could truly say, "to provide support for a lot of hungry politicians and still more for a whole army of their fellows, or minions." The ideal aim of a true representative government; "a government of the people, by the people and for the people" scarcely ever enters, and still more seldom fills, the brain of the average American politician. Science scarcely finds any support in this country either from the central or State Governments. Medical Science absolutely none. Scientific work in this country is simply the result of the sporadic endeavors of individuals; never of Government support as in Continental countries. The reason for this is to be sought in the total unfitness of the average American representative for the position he is elected to occupy. It is a purely American idiosyncrasy to suppose that men of general worldly ability and success have either the general or special education to enable them to legislate logically upon technical questions. Bigoted and unreflecting adherence to party principles without regard to independence of mind and faithfulness to national interests are the standard requisites sought in an American politician. Our Congressmen fail in comprehending that their work is of a national and not local character. What their local party constituency think should be a matter of little importance.

We see this unfitness of our Congressmen fitly illustrated in their deportment to the National Board of Health. Not one in fifty of them have general education and breadth of mind enough to comprehend the work that should be required of such a body. We see it again exemplified in their deportment with reference to matters strictly veterinary in their nature. We have before our eyes the farce of two Veterinary Powers in connection with the Government; one as a Treasury Cattle Commission, the other as a Veterinarian attached to the Agricultural Department. Nothing but jealousy between the departments, corresponding to that between the Board of Health and the Army Hospital Service, must be the natural result. The utter incompetency of the heads of these two departments with reference to the work of such Veterinary Powers, is shown by the work they are trying to do. The Government gives its support to quackery and empiricism and general incompetency by appointing on its Treasury Cattle Commission a non-educated, non-graduated empiric. No Government has a right to do this. The duty of a Government is to support the highest and best, and there are plenty of graduated and educated men in the country from whom a proper selection could be made. Further, the work of the commission is narrowed down to such contagious animal diseases, and none others, as have attracted the attention of Foreign Governments with reference to exported animals, chiefly cattle ; our domestic interests are of little account either to the Government or the Commission. No attempt is made to increase our knowledge with reference to the prevalence of disease in general and contagio-infectious disease in special among our domestic animals. The veterinary profession of the country is utterly neglected, and no demand has ever been made upon it, to report to the Government the results of each year's practical experience among our animals. The Veterinarians who have thus far been so fortunate, or unfortunate, as to receive Government appointments, state or central, have all shown but one desire ; viz.: to make themselves very prominent, and to make no use of the profession, save in such cases as they could find mere minions, or mouthpieces among its members. One government Veterinarian was so incompetent and so afraid some one would "steal his thunder" that he took an M. D. with him to Europe to make autopsies which he was unable to perform. This amounted to the same thing as telling the country, in his official capacity, that there was no Veterinarian in the country fitted to do such work. Some could be easily found, however, capable of instructing this Government Veterinarian and his medical assistant in this branch of work.

There is no more need of a Treasury Cattle Commission and an Agricultural Department Veterinarian than there is for a cow to have five legs ; one is superflous. This Agricultural Veterinarian is supposed to repeat the work of Pasteur, Koch and others, in Europe. This is Yankee effrontery. With the laboratories and means at their command it is absolute folly for us to try to do anything in the same line, until we can supply men with the same. What we need to do first, is to really know what diseases we have here ? How much they are extended ? What are the climatic, telluric, transport and other influences which play a part in their generation and extension. What we want at Washington is not only a man, or men, as Veterinarians, of education, but having a genius for the organization and planning of work of the very highest order ; a man who can not only work himself, but has that rare ability to make work for others and get it out of them, without letting himself be too prominent. No man can tell, and the present

Government Veterinarians are not trying to find out the extent to which glanders has extended among our horses; yet it is surely greater than the lung-plague among our cattle. We do not know anything about the extent of anthrax (or anthracoid diseases) among our animals; yet we read of "new diseases" continually appearing, which ignorance fails to recognize, but one as old as the history of man. Have we no Scab among our sheep? What extent does parasitic mange have among our dogs? How many cases of true Rabies take place in a year, and what is the damage caused by rabid dogs as well as non-rabid dogs among our sheep? To what extent does Tuberculosis prevail among our cattle?

The *cowardice*, prevarication, and incompetency of our Government is, however, better shown by its deportment toward the Trichinæ question than any other. To be sure we did all we could about Pleuro-pneumonia; first we had next to none at all, then a very few cases, and finally had to admit we had considerable; some ten or fifteen million dollars worth that is all. Then some one had the audacity to assert we had Trichinæ in our pork. Was ever anything so absurd! "There is no Trichinæ in American pork," says a Government circular sent to Foreign Governments, which assertion was known to be a lie and proven to be such.

Clause 8 and 9 of this wonderful Government circular read as follows:

8. "That the percentage of American hogs infected with trichinæ is, *in all probability, by reason of the superiority of the breed, and feeding, much less than that among the hogs of any other country.*"

(*a*) When a government through any of its departments makes a statement with reference to any question of so grave importance as *trichinæ* in our pork, it should have some stronger grounds than a "*probability*" upon which to support its assertions.

There are no records of the Government ever having authorized any examination of American hogs for trichinæ.

(*b*) Who ever heard of the *breed* of hogs having any influence upon trichinæ-infection? Who was the learned informant of our Government in this matter?

Superiority of feeding, so far as grain is concerned, has but little influence upon trichinæ-infection, if any at all, so long as hogs are allowed to root in refuse or run at large.

Such assertions can not be justifiably made until we know the *original source from which hogs derive trichinæ.*

With reference to "corn-feeding," Dr. Jansen T. Payne * makes some assertions which are of interest, but equally ridiculous with the foregoing, and show very little study of the trichinæ question. He says, of the hogs he examined at New Orleans: "All the hogs infected with trinchinæ ARE CORN-FED ANIMALS; *no mast-fed animals were found to be infected,*" which is as much as saying *that corn-feeding is a cause of trinchiniasis*—an assertion, as an investigator, I should be ashamed to have to father. Wild hogs that are not corn-fed have been found infected with trichinæ.

9. "That freedom from trichiniasis of the *two great pork-consuming centers of the West,* Chicago and Cincinnati, furnishes *the strongest possible evidence* of the purity of American pork. In Chicago, of 40,000 deaths, with causes reported, for a series of years, *only two* were from trichiniasis. During the same time NONE were reported in Cincinnati."

(*c*) Does the author mean that the people of these two cities consume more pork than those of New York, or the packing-houses?

(*d*) The consumption of pork as food bears no relation whatever to the number of cases of trichiniasis among people. One might almost be justified in saying *that the consumption of known trichinous pork did not.*

*The way it is eaten does, however. Well cooked or not,* that is the question.

Clause 10 indorses the last assertion:

"The reported cases of trichiniasis among human beings have resulted from eating uncooked pork," etc.

Hundreds of cases of trichinous infection are either so slight as to escape the attention of the doctors, or do not cause inconvenience enough to necessitate calling a doctor, or, as will be shown, if severe, are mistaken for something else. The tone of the government argument is false from the bottom; as

* Ninth Annual Meeting of American Public Health Association.

to eating *uncooked pork*, many slurs are made at our German citizens in this regard, while we, of English descent, fail to see that carnivorism and its results are common to all people ; the German indulges in raw, spiced, or smoked pork, and from it derives trichiniasis ; the American eats raw or uncooked beef, and derives a tape-worm. Comment is unnecessary, the only difference being, the German runs a greater risk of being sick or losing his life ; the act is the same.

The following examination of American Hogs was made by me and had been published in the Massachusetts Board of Health Reports long before the government circular was written :

*In making these examinations, a stump of the pillars of the diaphragm was invariable used, each piece representing one hog. But three microscopic specimens were made from each piece—a rule I invariably adhere to ; hence, if there is any error in my figures, it is in favor of the hogs.*

1879.

| LOT. | Number examined. | Non-trichinous. | Trichi-nous. | LOT. | Number examined. | Non-trichinous. | Trichi-nous. |
|---|---|---|---|---|---|---|---|
| 1 | 47 | 44 | 3 | 12 | 300 | 275 | 25 |
| 2 | 48 | 46 | 2 | 13 | 201 | 188 | 13 |
| 3 | 72 | 62 | 10 | 14 | 192 | 187 | 5 |
| 4 | 60 | 56 | 4 | 15 | 200 | 184 | 16 |
| 5 | 226 | 210 | 16 | 16 | 257 | 252 | 5 |
| 6 | 192 | 179 | 13 | 17 | 238 | 225 | 13 |
| 7 | 100 | 96 | 4 | 18 | 163 | 154 | 9 |
| 8 | 81 | 80 | 1 | 19 | 26 | 25 | 1 |
| 9 | 95 | 94 | 1 | 20 | 12 | 11 | 1 |
| 10 | 93 | 89 | 4 | | | | |
| 11 | 98 | 90 | 8 | | 2,701 | 2,547 | 154 |

Proportion, 1 : 17.154.

From the same source as the above :

1881.

| LOT. | Number examined. | Non-trichinous. | Trichi-nous. | LOT. | Number examined. | Non-trichinous. | Trichi-nous. |
|---|---|---|---|---|---|---|---|
| 1 | 127 | 120 | 7 | 10 | 125 | 118 | 7 |
| 2 | 130 | 127 | 3 | 11 | 127 | 122 | 5 |
| 3 | 153 | 150 | 3 | 12 | 122 | 118 | 4 |
| 4 | 120 | 115 | 5 | 13 | 124 | 118 | 6 |
| 5 | 124 | 123 | 1 | 14 | 100 | 100 | 0 |
| 6 | 100 | 99 | 1 | 15 | 122 | 115 | 7 |
| 7 | 119 | 113 | 6 | 16 | 120 | 114 | 6 |
| 8 | 127 | 123 | 4 | | | | |
| 9 | 160 | 152 | 8 | | 2,000 | 1,929 | 71 |

Proportion, 1 : 28.

From another source :

| LOT. | Number examined. | Non-trichinous. | Trichinous. | LOT. | Number examined. | Non-trichinous. | Trichinous. |
|---|---|---|---|---|---|---|---|
| 1 | 129 | 120 | 9 | 11 | 93 | 93 | 0 |
| 2 | 130 | 123 | 7 | 12 | 128 | 125 | 3 |
| 3 | 140 | 130 | 10 | 13 | 112 | 110 | 2 |
| 4 | 105 | 102 | 3 | 14 | 124 | 120 | 4 |
| 5 | 73 | 71 | 2 | 15 | 81 | 80 | 1 |
| 6 | 130 | 125 | 5 | 16 | 84 | 80 | 4 |
| 7 | 119 | 115 | 4 | 17 | 120 | 117 | 3 |
| 8 | 127 | 120 | 7 | 18 | 59 | 57 | 2 |
| 9 | 132 | 130 | 2 | | | | |
| 10 | 182 | 175 | 7 | | 2,068 | 1,993 | 75 |

Proportion, 1 : 27.

From a third source :

| LOT. | Number examined. | Non-triehinious. | Trichinous. | LOT. | Number examined. | Non-trichinous. | Trichinous. |
|---|---|---|---|---|---|---|---|
| 1 | 105 | 105 | 0 | 13 | 121 | 121 | 0 |
| 2 | 45 | 45 | 0 | 14 | 103 | 100 | 3 |
| 3 | 65 | 64 | 1 | 15 | 76 | 75 | 1 |
| 4 | 80 | 78 | 2 | 16 | 102 | 100 | 2 |
| 5 | 61 | 60 | 1 | 17 | 130 | 124 | 6 |
| 6 | 63 | 60 | 3 | 18 | 130 | 125 | 5 |
| 7 | 96 | 92 | 4 | 19 | 131 | 128 | 3 |
| 8 | 100 | 99 | 1 | 20 | 122 | 120 | 2 |
| 9 | 100 | 99 | 1 | 21 | 85 | 84 | 1 |
| 10 | 98 | 96 | 2 | | | | |
| 11 | 90 | 86 | 4 | | 2,004 | 1,959 | 45 |
| 12 | 101 | 98 | 3 | | | | |

Proportion, 1 : 44.

### RÉSUMÉ.

| | No. of hogs examined. | Trichinous. | Per cent. of infection. |
|---|---|---|---|
| 1879 | 2,701 | 154 | 1:17 |
| 1881, First source | 2,000 | 71 | 1:28 |
| 1881, Second source | 2 068 | 75 | 1:27 |
| 1881, Third source | 2,004 | 45 | 1:44 |
| Total | 8,773 | 345 | 1:25 |

These figures certainly do not serve to support the words of the State Department, that there are "*less trichinæ in American pork than that of any other country.*" They do speak in no uncertain terms, that our Government has an imperative duty which it owes a large national interest, until the original source whence swine become invaded be discovered.

It has been said already that but about fifty of these 8,773 hogs were bought at Chicago; hence, were Western hogs, though killed and examined at Boston. They were purchased at the same yards from which the Chicago packing-houses obtain the pork which our State Department declared to be so "*free from trichinæ.*"

Further, the percentage of infection of the hogs from the three different sources is interesting, but not easy of explanation, but no more varying than that of those examined in 1879 and 1881 from the same source.

This variation in the per cent. of infection between those examined in 1879 and 1881 called forth the following remarks from Dr. Loring, the present Commissioner of Agriculture:

"A veterinarian of New England informed me on the 14th of April last that he had examined portions from 2,701 Western hogs, obtained in Boston, 154 of which he found infected, i. e., one case to each $17\frac{54}{100}$ hogs examined. He tells me that he will make a statement to this meeting that he has examined portions of 8,773 Western animals, and has found one case to every 25 animals. *You will see that there is a great difference between his first (April) examination and this one, and his result is so greatly different from the English examination of our hogs, above mentioned, and so much above any known percentage among animals of every other country, that I cannot but entertain doubts of the value of his examination.*"*

The English examination spoken of reads as follows:

"*The inspectors of the Veterinary Department examined two hundred and seventy-nine separate portions of swine's flesh, which were sent from Liverpool, and detected living trichinæ in three specimens*" (1:93).

Were Dr. Loring anything but a physician, such criticism as the foregoing might be passed as unworthy of notice, but from a physician, who should have some knowledge of the variation of medical statistics, we should expect some other argument than *empty words.*

First, as to the discrepancy spoken of between the results of my examinations, made about a year apart: it is not greater than that between any two lots taken at random in the same examination, nor so great as between very many lots examined on two consecutive days; as, for instance, in my series of 1881, Lot 14 (source the same as in 1879) was 100 pieces, of which NONE were infected, while of Lot 13, 124 pieces, *six were trichinous.*

In two different epidemics of small-pox, the number of

* See letter to Health Congress, previously referred to.

deaths is never the same, or even the number of cases. Are we then to say a later invasion is not small-pox, because the number of cases or deaths is less or more than in a previous? I never for a moment expected similar results, and should have been as pleased to find none as any one in the country.

With reference to the English examination, 1:93, *it is greater by far than the percentage of infection found in the hogs of any other country, and greater than I found in some lots examined by me; for instance, Lots* 1, 2, 3, 4 *of my third series,* 1881, *contained, respectively,* 105, 45, 65, *and* 80 *specimens, respresenting* 295 *hogs, of which three were trichinous,* 1:98. Further, we do not know the parts the English examined; had they been pillars of the diaphragm, the per cent. might have been greater.

As to the *correctness* of my results, I will simply say that Dr. Folsom, of the Massachusetts Board of Health, went over a large part of those examined of 1879, and that competent physicians and a gentleman whom I educated to work with me, whom I can call upon for testimony, continually revised my other specimens as I examined them.

Again, if the Commissioner of Agriculture doubts my results, let him send a competent man or men here and examine with me the same specimens, be it one or ten thousand, and I venture to say we shall find a percentage of infection *larger than that reported in any other country, and large enough to satisfy any one.*

Further, the Germans, well doubt the figures of their own examinations, as, from the Prussian statistics, we see the percentage of infection is steadily augmenting.

I wish now to refer to the report of Dr. Jansen T. Payne,* which I have before alluded to, from which I quote the following:

"The method of conducting the researches was as follows: 'The examples procured one afternoon were examined the following day by the aid of a good microscope, capable of magnifying objects *two hundred diameters. A low power was found to give greater satisfaction than a higher one could have done, and observers in this field would do well to bear this in mind. When it is taken into account that each of the specimens had to be separated*

* Report of the American Public Health Association, 1881.

*into minute shreds before they were placed upon the stage of the miscroscope, and consider the number of fibres examined in such cases'*" (he examined in all 21,600 specimens from 5,400 hogs), "'*it will readily be perceived that it is impossible to make anything like an accurate guess as to the whole number of pieces of muscle-fibre examined.*

Result: Number examined, 5,400; trichinous, 22.

"*By this series of examinations, it has been ascertained that Southern-bred hogs are free from trichinæ.*"

If there is anything I dislike to do, it is to criticise the work of another observer; but one would like to know if *two hundred diameters* is considered a *low* power. For myself, when looking for trichinæ, should I use such a power, I should not expect to find many trichinæ, but boa-constrictors; in fact many would escape me. The male trichina measures one eighth of an inch in length—magnified two hundred diameters, what would one have?

Again dividing specimens into shreds may be highly technical, but eminently unpractical; for with crush-specimens one can easily recognize the parasite, and it is done quickly, while in this way, and such a high power as two hundred diameters, one would be sure to miss many.

I doubt the statement that "*Southern hogs are free from trichinæ*" as much as I do that "*corn-feeding*" *has anything to do* with trichiniasis.

My observations have been made, but not of Massachusetts hogs, at Boston, which is not the place for such work, except so far as the obtaining of the per cent. of infection of hogs which have come directly from the breeder or fattener to the packer. Individual lots could be examined, and traced back to the source whence they came. If found highly infected, it would be easy to go to such places and make all manner of examinations—of remaining hogs, the earth, worms, grubs, etc. *Some unknown living thing lodges trichinæ before they enter the porcine organism.* The questions are: *What is it? Where is it? What are its modes of life?*

Bluffing the attempt to write down an existing fact will only end in ridicule to this Government; *research*, untiring and unceasing, is the only "*cure*;" cost what it will, it will pay far better than governmental lies.

American hogs are much more trichinous than European.

The diseases of our cattle are undoubtedly of great importance, but they bear no comparison with trichinasis of our hogs either from a monetary or sanitary point of view.

We have said enough to indicate the work our Government has before it in reference to the diseases of our animals. As Veterinarians we have but one work to do, and that is to keep hammering at the door of Congress until it recognizes the fact that such diseases do exist, and that the only way to prevent them is to recognize the fact that we have a Veterinary profession in this country, small in numbers but no less earnest in purpose than that of any other country.

